# Development of feeding systems and strategies of supplementation to enhance rumen fermentation and ruminant production in the tropics

**DOI:** 10.1186/2049-1891-4-32

**Published:** 2013-08-27

**Authors:** Metha Wanapat, Sungchhang Kang, Sineenart Polyorach

**Affiliations:** 1 Tropical Feed Resources Research and Development Center (TROFREC), Department of Animal Science, Faculty of Agriculture, Khon Kaen University, Khon Kaen 40002, Thailand; 2 Department of Animal Science, Faculty of Natural Resources, Rajamangala University of Technology-Isan, Sakon Nakhon Campus, Phang Khon, Sakon Nakhon 47160, Thailand

**Keywords:** Feed resources, Feeding system, Methane, Plant secondary compounds, Ruminants

## Abstract

The availability of local feed resources in various seasons can contribute as essential sources of carbohydrate and protein which significantly impact rumen fermentation and the subsequent productivity of the ruminant. Recent developments, based on enriching protein in cassava chips, have yielded yeast fermented cassava chip protein (YEFECAP) providing up to 47.5% crude protein (CP), which can be used to replace soybean meal. The use of fodder trees has been developed through the process of pelleting; *Leucaena leucocephala* leaf pellets (LLP), mulberry leaf pellets (MUP) and mangosteen peel and/or garlic pellets, can be used as good sources of protein to supplement ruminant feeding. Apart from producing volatile fatty acids and microbial proteins, greenhouse gases such as methane are also produced in the rumen. Several methods have been used to reduce rumen methane. However, among many approaches, nutritional manipulation using feed formulation and feeding management, especially the use of plant extracts or plants containing secondary compounds (condensed tannins and saponins) and plant oils, has been reported. This approach could help todecrease rumen protozoa and methanogens and thus mitigate the production of methane. At present, more research concerning this burning issue - the role of livestock in global warming - warrants undertaking further research with regard to economic viability and practical feasibility.

## Introduction

Animals have been an important component in integrated crop-livestock farming systems in developing countries. In a diversified role, they produce animal protein food, draft power and farm manure as well as ensuring social status and enriching people’s livelihoods [1]. As the world population is expected to increase from 6 billion to about 8.3 billion in the year 2030 at an average growth rate of 1.1% per yr, it is essential to be prepared to produce sufficient food for the increased population based on locally available resources especially in the developing countries. The consumption of animal food was 10 kg/yr in the 1960s increasing to 26 kg/yr in 2000 and is expected to be 37 kg/yr by 2030 [2,3]. Livestock production, in particularly buffalo, cattle and small ruminants, is an integral part of food production systems, making important contributions to the quality and diversity of the human food supply as well as providing other valuable services such as work and nutrient recycling. Large increases in per capita and total demand for meat, milk and eggs are forecast for most developing countries for the next few decades [4]. In developed countries, per capita intakes are forecast to change slightly, but the increases in developing countries, with their larger populations and more rapid population growth rates, will generate a very large increase in global demand. Most importantly, the conversion of materials inedible for humans, such as roughage, tree fodder, crop residues and by-products, into human food by ruminant animals will continue to serve as an important function of animal agriculture. However, since much of the projected increase is expected to come from pork, poultry and aquaculture production, and especially from species consuming diets high in forage carbohydrate, meeting future demand will depend substantially on achievable increases in cereal yields. Therefore, there are opportunities and challenges for researchers to increase animal productivity through the application of appropriate technologies, particularly in production systems, nutrition and feeding. Wanapat [5] and Devendra and Leng [6] have emphasized the utmost importance of using local feed resources as the key driving force to increase the productivity of animals in Asia.

Global warming is a highly important issue which affects the environment and livestock production. Total emissions of greenhouse gases (GHG) from agriculture, including livestock, are estimated to be between 25% and 32%, depending on the source [7,8] and on the proportion of land conversion that is ascribed to livestock activities. Interestingly, Goodland and Anhang [9] reported that livestock production and its by-products are responsible for at least 51 percent of global warming gases, accounting for at least 32.6 billion tons of carbon dioxide per yr. Carbon dioxide provides most GHG (55-60%) followed by methane (15-20%). Therefore, livestock is one source of methane production through fermentation in the rumen. Gas emissions from the livestock sector are estimated at between 4.1 and 7.1 billion tons of CO_2_ equivalents per yr, equating to 15-24% of total global anthropogenic GHG emissions [10].

Tropical plants normally contain a high to medium content of secondary compounds such assaponins and condensed tannins, which have been shown to exert a specific effect against rumen protozoa while leaving the rest of the rumen biomass unaltered [11]. Numerous studies have determined the effects of feeding ruminants with saponin-rich plants such as *Enterelobium cyclocarpum, Spinadus saponaria, Sapindus rarak, Sesbania sesban, Quillajasaponaria, Acaciaauriculoformis* and *Yucca schidigera*[11,12]. Results have indicated that saponins have a strong anti-protozoal activity and could thus serve as an effective defaunating agent for ruminants due to their detergent action [13]. Numerous studies [14-16] have recently reported the impact of livestock on global warming and suggested approaches to mitigate rumen methane.

### Development of pelleted feeds

Pelleted feeds have been used successfully for fish and animals including non-ruminant and ruminant animals, fish and shrimp. The advantages of pelleted feeds include: (1) preventing selective feeding on those ingredients in the formulation which are more palatable and thus more desirable to the animal; (2) preventing the separation of constituents in animal feeds due to varying size and density; (3) providing higher bulk density, which has advantages both for shipping and handling, resulting in maximum load efficiency and reduced storage requirements; and (4) improving nutrient utilization and so increasing the feed conversion rate. Pelleting also improves the acceptability, density and keeping quality of feedstuffs [17]. Generally, pelleted feeds are produced in an extrusion-type thermoplastic melding operation in which finely divided particles of a feed ration are formed into compact, easily-handled pellets. Binder additives may be used to improve the strength and shelf-life of pellets and to reduce the release of fines during the pelleting process. Preferably, nutritive binder additives are used which also provide essential recognized nutrients such as magnesium, calcium, potassium and/or sulfur to the feed.

**Figure 1:**
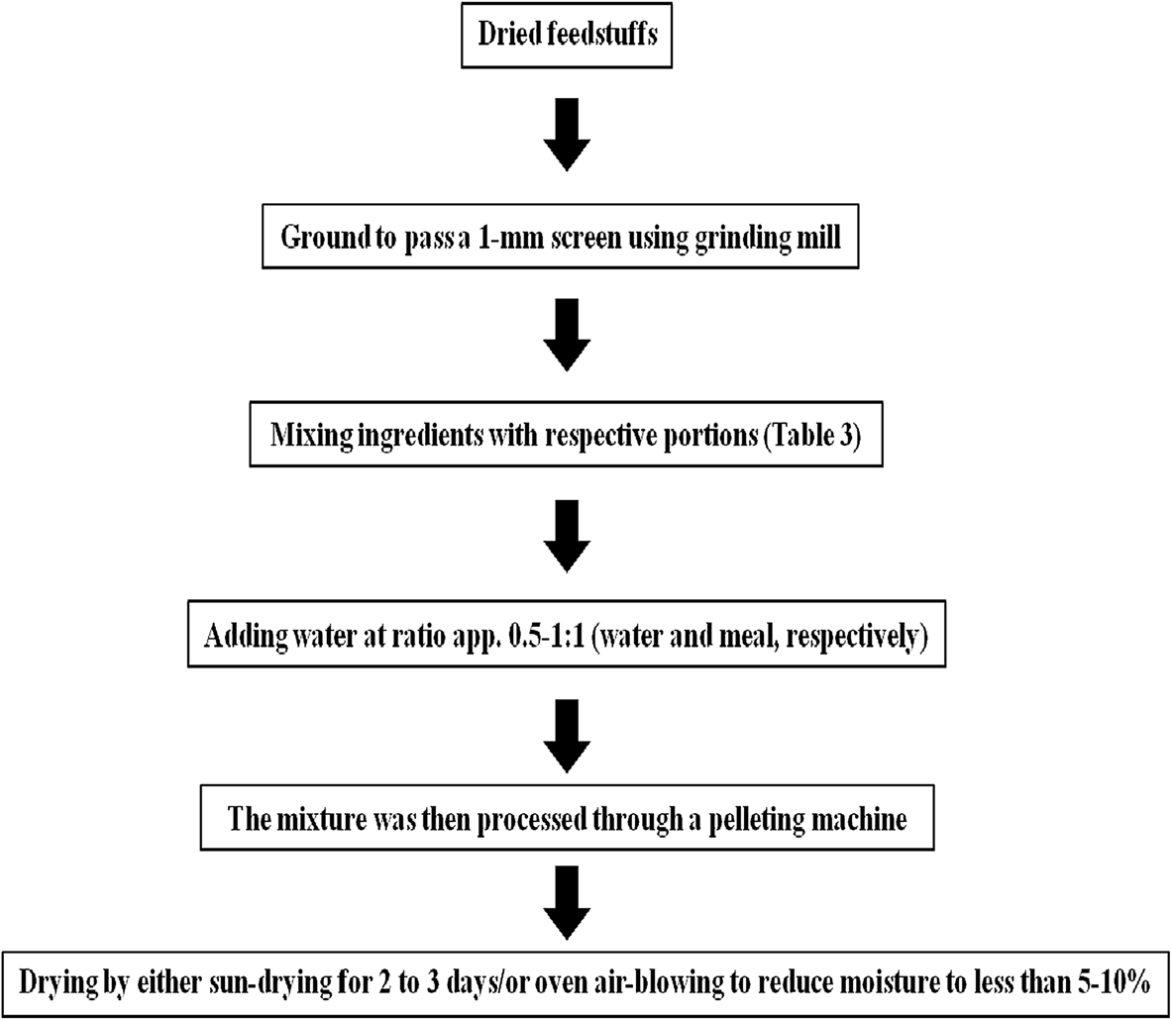
Processing chart for pelleting the products (Mago-pel, Maga-lic, Maga-ulic, LLP, MUP and SWEPP).

Recently, scientists have been interested in pelleting local feed resources and agricultural cropresidues, such as mangosteen (*Garcinia mangostana*) peel, mulberry (*Morus alba*), Leucaena (*Leucaena leucocephala*), sweet potato (*Ipomoea batatas*) vine, to improve the nutritive value and its utilization. Pellet products such as Mago-pel (mangosteen peel pellet), Maga-lic (mangosteen peel with garlic powder pellet), Maga-ulic (mangosteen peel pellet with urea and garlic powder), LLP (leucaena leaf pellet), MUP (mulberry leaf pellets) and SWEPP (sweet potato vine pellet with 10% urea) have been prepared following the steps shown in Table 1 and Figure 1.

**Table 1:** Feed ingredients and chemical composition of Mago-pel, Maga-lic, Maga-ulic, LLP, MUP and SWEPP

**Items**	**Mago-pel**	**Maga-lic**	**Maga-ulic**	**LLP**	**MUP**	**SWEPP**
Ingredients	% of dry matter					
Mangosteen peel powder	98.5	93.5	91.5	-	-	-
Garlic powder	-	5	5	-	-	-
Leucaena leaf meal	-	-	-	81	-	-
Mulberry meal	-	-	-	-	82	-
Sweet potato vine	-	-	-	-	-	81.5
Cassava starch	0.5	0.5	0.5	0.5	0.5	0.5
Urea	-	-	0.2	10	10	10
Molasses	1	1	1	5	4.5	5
Sulfur	-	-	-	1	1	1
Mineral mixture	-	-	-	1	1	1
Salt	-	-	-	1	1	1
Chemical composition						
Dry matter	93.3	93.1	92.7	92.9	92.3	95.6
	% of dry matter					
Organic matter	96.5	96.4	96.5	91.3	88.2	81.4
Crude protein	21.2	21.5	22.1	42.2	48.7	40.5
Neutral detergent fiber	57.3	57.2	57	44	20.4	33.1
Acid detergent fiber	48.6	48.2	48.3	20	14.5	27.8

*Abbreviations*: *Mago-pel* mangosteen peel pellet, *Maga-lic* mangosteen peel with garlic powder pellet, *Maga-ulic* mangosteen peelpellet with urea and garlic powder, *LLP* leucaena leaf pellet, *MUP* mulberry leaf pellets, *SWEPP* sweet potato vine pellet with 10% urea.

Huyen et al. [18] and Tan et al. [19] have reported that supplementation with mulberry leaf pellets (MUP) improved nutrient digestibility and rumen fermentation. MUP could be used as a protein source to improve rumen efficiency and production especially supplementation at 600 g/d for beef cattle when fed on low-quality roughage such as rice straw. Norrapoke et al. [20] showed that the combined use of concentrates containing 16% CP with Mago-pel at 300 g/hd/d resulted in changes in rumen fermentation and microbial population and an improvement in milk production in lactating dairy crossbreds. Manasri et al. [21] reported that supplementation with Maga-lic at 200 g/hd/d improved ruminal fermentation, especially increasing the proportion of propionate and reducing methane gas production in beef cattle steers. Furthermore, Trinh et al. [22] compared non-supplemented and pellet-supplemented groups of beef cattle (Mago-pel, Maga-lic and Mago-ulic at 200 g/hd/d) It was found that total dry matter intake (DMI) and digestibility of DM and CP were not significantly affected by pellet supplementation when compared with the control group (*P* > 0.05). In addition, the acetate content, the acetate:propionate ratio, the protozoa population and methane production were all reduced, whereas the propionate production and bacterial population increased in the pellet-supplemented group and were highest in the Maga-ulic-supplemented treatment. The Maga-ulic supplemented treatment also provided the highest level of microbial protein synthesis when compared with the other treatments. Hung et al. [23] reported that LLP supplementation significantly increased rice straw intake and total intake. There was also an increase in the population of fungal zoospores, amylolytic bacteria, proteolytic bacteria and cellulolytic bacteria with an increasing level of LLP supplementation while the population of rumen protozoa decreased. The population of total bacteria and the three predominant cellulolytic bacteria increased when the level of LLP supplementation increased; meanwhile, the population of methanogenic bacteria decreased. Supplementation with LLP resulted in an improvement in nitrogen balance and microbial nitrogen supply. Recently, Phesatcha and Wanapat [24] revealed that SWEPP was a good source of protein supplement improving apparent digestibility, rumen fermentation, and milk yield in lactating dairy cows. A summary of the experimental data above is presented in Table 2.

**Table 2:** Effect of of Mago-pel, Maga-lic, Maga-ulic, LLP, MUP, SWEPP on DMI, digestibility, rumen volatile fatty acid (VFA) production and ruminal microorganisms

**Pelleting**	**Suppl.**	**Animal**	**DMI**	**Dig.**	**VFA**			**CH**_**4**_	**MPS**	**Prot.**	**Reference**
					**C2**	**C3**	**C4**				
MUP	600 g/hd/d	Buffalo	↑	↑	↓	↑	↑	↓	nd	↓	[18]
MUP	600 g/hd/d	Buffalo	↑	nd	nd	nd	nd	nd	↑	nd	[19]
Mago-pel	300 g/hd/d	Dairy cow	nc	nc	nc	nc	nc	nc	↑	↓	[20]
Maga-lic	200 g/hd/d	Dairy steer	nc	↑	↓	↑	nc	↓	nd	↓	[21]
Maga-ulic	200 g/hd/d	Dairy steer	nc	↑	↓	↑	nc	↓	↑	↓	[22]
LLP	450 g/hd/d	Buffalo	↑	nd	nd	nd	nd	nd	↑	↓	[23]

*Abbreviations*: *MUP* mulberry leaf pellet, *Mago-pel* mangosteen peel pellet, *Maga-lic* mangosteen peel and garlic pellet, *Maga-ulic* mangosteen peel, garlic and urea pellet, *LLP* leucaena leaf pellet, *VFA* volatile fatty acid, *C2* acetic acid, *C3* propionic acid, *C4* butyric acid, *CH*_*4*_ methane production, increase (↑), decrease (↓) from control group, *nd* not determined, *nc* no change.

### Yeast fermented cassava chip protein (YEFECAP)

**Figure 2:**
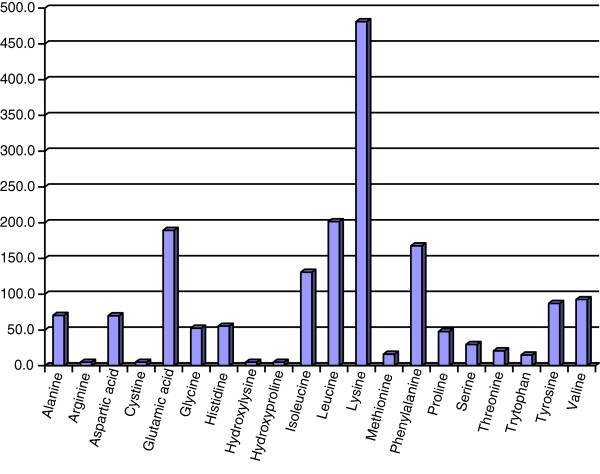
**Amino acid profile of YEFECAP products (mg/100 g of YEFECAP).** Source: Polyorach et al. [26].

Cassava chip and other forms of cassava root can be successfully fermented with yeast (*Saccharomyces cereviceae*) to obtain a final product with high CP and a relatively high profile of amino acids [25,26]. The amino acid profile of YEFECAP is presented in Figure 2, showing a high level of lysine, glutamic acid, leucine and phenylalanine. Supplementation with YEFECAP to replace soybean meal in concentrates for lactating dairy cows resulted in a good performance in milk yield (15.7 kg/d) [27].

**Figure 3:**
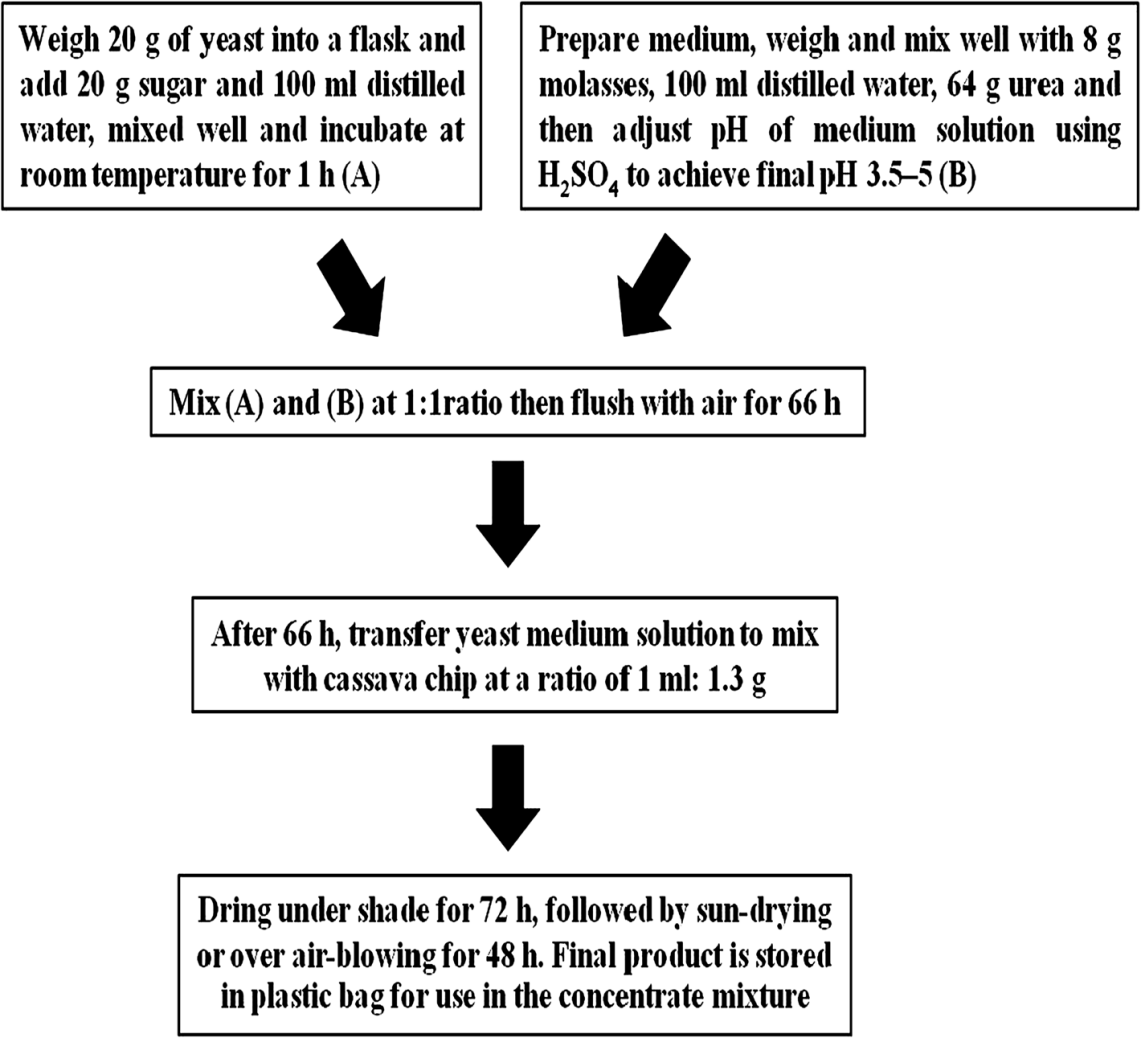
**Process chart for yeast fermented cassava chip products (YEFECAP) preparation.** Polyorach et al. [29].

Dietary yeast can be used as a ruminant feed especially *Saccharomyces cerevisiae* because the yeast cell contains useful nutrients for ruminant feed, especially a high lysine content (7.6 ± 0.7 g/16 g N) [26,28,29]. Moreover, the addition of yeast to the ruminant diet can not only improve the rumen environment but also enhance microbial activities (especially cellulolytic activities so that they increase fiber digestion, reduce lactate accumulation and the concentration of oxygen in rumen fluid and improve the utilization of starch [30,31]. In addition, *S. cerevisiae* could also stimulate DM intake and productivity in growing and lactating cattle [32] and improve microbial protein synthesis and milk production in dairy cows [33,34]. However, Desnoyers et al. [35] reported that the highly variable effects of live *S. cerevisiae* cultures could be associated with the ratio of forage to concentrate used. Cassava chip is an energy source with low crude protein, but when fermented with yeast can increase crude protein from 1-3% CP to 30.4% CP [36]. Recently, Polyorach et al. [26,29] reported that YEFECAP could be prepared with aCP level up to 47%. The YEFECAP was prepared according to the method of Polyorach et al. [29] as shown in Table 3 and Figure 3.

**Table 3:** Chemical composition of yeast fermented cassava chip protein (YEFECAP)

**Chemical composition**	**YEFECAP**
Dry matter	90.6
	% of dry matter
Organic matter	97.2
Crude protein	47.5
Ether extract	7.9
Neutral detergent fiber	6.1
Acid detergent fiber	4.3

Source: Polyorach et al. [26].

The beneficial use of YEFECAP has been evaluated by Boonnop et al. [37] and Wanapat et al. [27,38]. Boonnop et al. [37] studied the effects of replacing soybean meal with YEFECAP on rumen ecology and nutrient digestibility in dairy crossbred steers. It was found that YEFECAP could replace soybean meal completely and was beneficial to cattle in terms of the efficiency of rumen fermentation, microbial protein synthesis, nitrogen retention and nutrient digestibility. Khampa et al. [39] reported that supplementation with YEFECAP could replace 75% of concentrate to improve ruminal fermentation efficiency and average daily gain and also reduce the cost of production in dairy heifers. Supplementation with YEFECAP could improve the population of bacteria and fungal zoospores, but decrease the population of *Holotrich* and *Entodiniomorph* protozoain the rumen of dairy steers [40]. Polyorach et al. [41] and Wanapat et al. [27,38] revealed that using YEFECAP to replace soybean meal at 0, 33, 67 and 100% CP could enhance milk yield, milk fat and milk protein with increasing YEFECAP level and was highest at a 100% level of replacement. Moreover, Wanapat et al. [38] compared four sources of protein in concentrate diets, soybean meal (SBM), cassava hay (CH), *Leucaena leucocephala* (LL) and YEFECAP in lactating dairy cows and found that CP digestibility was highest in CH- and YEFECAP-supplemented groups. Propionic acid content was highest in cows receiving CH and YEFECAP, while populations of ruminal fungi, proteolytic and cellulolytic bacteria were highest with YEFECAP supplementation. Milk fat and milk protein content significantly increased in cows fed with CH and YEFECAP (*P* < 0.05). Based on these studies, YEFECAP can be practically prepared and used as an alternative protein source in ruminant feeding (Table 4).

**Table 4:** Effect of using YEFECAP as a protein source in ruminants on DMI, digestibility, rumen volatile fatty acid (VFA) production, ruminal microorganisms, and milk production in various studies

**Animal**	**DMI**	**Dig.**	**TVFA**	**C2**	**C3**	**C2:C3**	**Bact**	**Prot**	**Fung**	**MSP**	**Milk**			**Reference**
											**Yield**	**Fat**	**Protein**	
Lactating dairy cows	↑	↑	↑	nc	↑	↓	↑	nc	↑	nd	↑	↑	↑	[27]
Dairy steers	↑	↑	↑	↓	↑	↓	↑	↓	↑	↑	nd	nd	nd	[37]
Lactating dairy cows	ns	↑	↑	nc	↑	nd	↑	↓	↑	nd	nc	↑	↑	[38]

*Abbreviations*: *DMI* dry matter intake, *TVFA* total volatile fatty acid, *C2* acetate, *C3* propionate, *C2:C3* proportion of acetate to propionate, *MPS* microbial protein synthesis, increase (↑), decrease (↓) from control group, *nd* not determined, *nc* no change.

Wanapat et al. [27,38] reported on a study using YEFECAP to replace soybean meal (SBM) in concentrate mixtures for early lactating cows. It was found that YEFECAP can completely replace SBM in concentrate mixtures for milking dairy cows while enhancing rumen fermentation, dry matter intake, nutrient digestibility, milk yield and composition. A summary of the above research data is shown in Table 5.

**Table 5:** Effect of YEFECAP as a protein source in concentrate mixtures on milk production, milk composition and economic return

**Items**	**Treatments**				**SEM**	**Contrasts**		
	**T1**	**T2**	**T3**	**T4**		**L**	**Q**	**C**
Production								
Milk yield, kg/d	13.5	14.0	14.5	15.0	0.27	**	ns	ns
3.5% FCM^1^, kg/d	13.7	14.7	15.9	17.1	0.49	**	ns	ns
Milk composition, %								
Protein	4.0	4.1	4.5	4.7	0.17	**	ns	ns
Fat	3.2	3.3	3.4	3.5	0.06	**	ns	ns
Lactose	4.5	4.6	4.6	4.7	0.07	ns	ns	ns
Solids-not-fat	8.2	8.4	8.4	8.5	0.29	ns	ns	ns
Total solids	12.3	12.7	12.8	13.0	0.78	ns	ns	ns
Milk urea N, mg/dL	14.8	12.5	12.3	12.0	0.58	*	ns	ns
Economic return, $US/hd/d								
Feed cost	2.5	2.6	2.6	2.7	0.14	ns	ns	ns
Milk sale	9.5	9.8	10.2	10.5	0.19	**	ns	ns
Profit	7.0	7.2	7.6	7.8	0.16	**	ns	ns

Source: Wanapat et al. [27].

*ns*, non-significance.

Level of replacement of soybean meal (SBM) by YEFECAP: at 0% (T1), 33% (T2), 67% (T3), 100% (T4).

Feed cost: concentrate *T1*, 0.38 $US/kg, *T2*, 0.37 $US/kg, *T3*, 0.37 $US/kg, *T4*, 0.35 $US/kg, *ULRS*, 0.07 $US/kg, *Milk price*, 0.7 $US/kg.

*L*, linear, *Q*, quadratic, *C*, cubic, *SEM*, standard error of the means; * *P*<0.05; ** *P*<0.01.

### Use of plant secondary compounds in methane reduction

**Figure 4:**
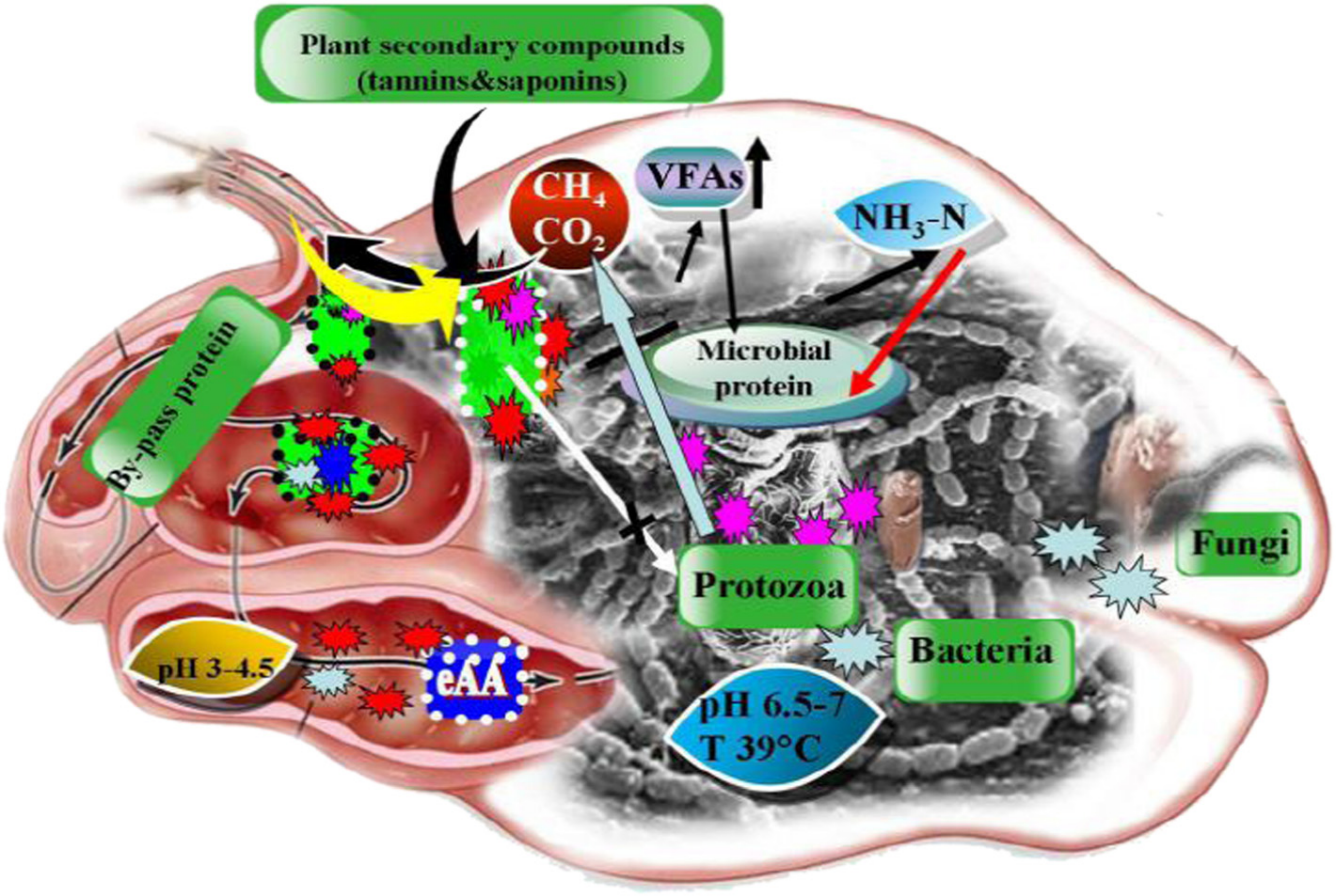
**Role of plant secondary compounds (condensed tannins and saponins) on rumen fermentation process **[1]**.**

Plant secondary compounds (condensed tannins and saponins) are important ruminant feed additives, particularly for a methane mitigation strategy because of their natural origin as opposed to chemical additives (Figure 4). Anti-methanogenic activity can be attributed to both condensed tannins and hydrolysable tannins. There are two modes of action of tannins in methanogenesis: a direct effect on ruminal methanogens and an indirect effect on hydrogen production due to lower feed degradation. There is also evidence that some condensed tannins (CT) can reduce methane emissions while reducing bloat and increasing amino acid absorption in the small intestine. Methane emissions are also commonly lower with higher proportions of forage legumes in the diet, partly due to lower fiber contact, a faster rate of passage and, in some cases, the presence of condensed tannins [42,43]. Supplementation with *Phaseolus calcaratus* hay (PCH) at 600 g/hd/d was beneficial for swamp buffaloes fed rice straw as a basal roughage, as it resulted in increased DM intake, reduced protozoal numbers and methane gas production in the rumen, increased N retention as well as improving the efficiency of rumen microbial CP synthesis [44]. Legumes containing condensed tannin (e.g. Lotuses) are able to lower methane (based on g/kg DMI) by 12-15% [42,45]. Also, some authors have reported that condensed tannins can reduce methane production by 13 to 16% (DMI basis) [46,47], mainly through a direct toxic effect on methanogens. More recently, Woodward et al. [47] carried out a similar trial with cows fed *Lotus corniculatus* and found that methane production were reduced,McAllister and Newbold [48] reported that extracts from plants such as rhubarb and garlic could also decrease methane emissions. However, there is little information on the effect of different saponins on rumen bacteria. In one study, Sirohi et al. [49] showed that plant secondary metabolites (PSM) at low concentrations could be used to manipulate rumen fermentation favorably. At an appropriate dose, saponins or saponin-containing plants have been shown to suppress the protozoal population, increase the bacteria and fungi population, the production of propionate, the partitioning factor, the yield and efficiency of microbial protein synthesis and to decrease methanogenesis, all of which improve performance in ruminants. Tannins, especially condensed tannins (CT), also decrease methane production and increase the efficiency of microbial protein synthesis. Plant extracts, rich in flavonoids, increase the degradation of cell wall constituents and also the yield and efficiency of microbial protein synthesis [49].

Saponins are natural detergents found in many plants. Interest has increased in using saponin-containing plants as a possible means of suppressing or eliminating protozoa in the rumen. Decreased numbers of ruminal ciliate protozoa may enhance the flow of microbial protein from the rumen, to increase the efficiency of feed utilization and decrease methanogenesis. Saponins are also known to influence both the composition and number of ruminal bacterial species through specific inhibition or selective enhancement of the growth of individual species. Saponins have been shown to possess strong defaunation properties both *in vitro* and *in vivo* which could reduce methane emissions [45]. Beauchemin et al. [42] recently reviewed the literature related to the effect of saponins on methane and concluded that although there is evidence for a reduction in methane from some sources of saponins, not all are effective [45]. While extracts of CT and saponins may be commercially available, their cost is currently prohibitive for their routine use in ruminant production systems. However, research is still required on the optimum sources of CT and saponins, the level of CT astringency (chemical composition) and the feeding methods and dose rates required to reduce methane and stimulate animal production.

Table 6 presents the data from both *in vitro* and *in vivo* trials using mangosteen peel powder (MP) with or without other sources on rumen fermentation. Based on these results, MP supplementation both for *in vitro* and *in vivo* trials significantly increased the production of total volatile fatty acids (*P* < 0.05), as well as propionate production, while acetate, butyrate production and the acetate:propionate ratio were significantly decreased (*P* < 0.05). Condensed tannins and saponins contained in MP could contribute to the above effects. Similar effects, especially regarding the acetate:propionate ratio, were found by Beauchemin and McGinn [50] while total volatile fatty acids were decreased. The effects of supplementation with MP on DM intake, digestibility and rumen methane production are reported in Table 7. These findings showed that MP supplementation did not affect DM intakes, while digestibility and rumen methane production (by estimation using volatile fatty acid concentration) were significantly decreased (*P* < 0.05). The effects of MP supplementation on the population of ruminal microorganisms are shown in Table 8. MP supplementation reduced rumen protozoa production remarkably, while the numbers of the predominant cellulolytic bacteria increased (*P* < 0.05). In addition, methanogen numbers tended to decrease. However, it was found that mangosteen peel powder significantly increased (*P* < 0.05) the cellulolytic bacteria population [51]. The condensed tannins and saponins present in the MP could influence such changes in the rumen.

**Table 6:** **Effect of mangosteen peel supplementation on rumen volatile fatty acid production in ruminants using *in vitro *and *in vivo *studies**

**Substrate**	**Level**	**Species**	**TVFA**	**C**_**2**_	**C**_**3**_	**C**_**4**_	**C**_**2**_**/C**_**3**_	**References**
*In vitro*								
MP	200 mg	Steer	+	+	+	─	─	[52]
*In vivo*								
MP	100 g/hd/d	Beef cattle	+	─	─	─	─	[52]
MP	200 g/hd/d	Dairy cows	+	─	+	+	─	[53]
MP	100 g/hd/d	Native cattle	+	─	+	─	─	[51]
MP	30 g/kg	Buffalo	+	─	+	─	─	[54]
MPP	200 g/hd/d	Beef cattle	+	─	+	─	─	[22]
MPP	300 g/hd/d	Dairy cow	+	─	+	─	─	[20]
Combination								
CO + MP	50 + 30 g/kg	Buffalo	─	─	+	─	─	[54]
MP + GP	9 + 1%	Beef cattle	+	+	+	─	─	[22]
MP + GP pellet	200 g/hd/d	Beef cattle	+	─	+	─	─	[22]

*Abbreviations*: *GP* garlic powder, *MP* mangosteen peel powder, *MPP* mangosteen peel pellet, *CO* coconut oil, + increased, ─ decreased.

**Table 7:** **Effect of mangosteen peel supplementation on intake, digestibility and methane production in ruminants using *in vitro *and *in vivo *studies**

**Substrate**	**Level**	**Species**	**DMI**	**Dig**	**CH4**	**References**
*In vivo*						
MP	100 g/hd/d	Beef cattle	+	+	─	[52]
MP	200 g/hd/d	Dairy cows	nc	+	─	[53]
MP	100 g/hd/d	Native cattle	nc	+	─	[55]
MP	30 g/kg	Buffalo	nc	─	─	[54]
MPP	200 g/hd/d	Beef cattle	nc	+	─	[22]
MPP	300 g/hd/d	Dairy cows	+	nc	─	[20]
Combination						
CO + MP	50 + 30 g/kg	Buffalo	nc	+	─	[54]
MP + GP	9 + 1%	Beef cattle	nc	+	─	[22]
MP + GP pellet	200 g/hd/d	Beef cattle	nc	+	─	[22]

*Abbreviations*: *GP* garlic powder, *MP* mangosteen peel powder, *MPP* mangosteen peel pellet, *CO* coconut oil, *nc* not changed.

+ increased, - ─ decreased.

**Table 8:** Effects of mangosteen peel powder supplement on population of rumen microbes

**Substrates**	**Level, g/h/d**	**Protozoa**	**Methanogens**	**RF**	**RA**	**FS**	**Species**	**References**
		**(+/−)**	**(+/−)**	**(+/−)**	**(+/−)**	**(+/−)**		
MP	100	−*	nd	nd	nd	nd	Beef cattle	[52]
MP	100	−	−	+*	+*	+*	Native cattle	[51]
MP	300	−*	nd	+	+	+	Dairy cows	[20]

*Abbreviations*: *MP* mangosteen peel powder, plus symbol, minus symbol increase or decrease from control group, *nd* not determined, *RF ruminococcus flavefaciens*, *RA ruminococcus albus*, *FS fibrobactor succinogenes* *P < 0.05, significantly different from control group.

+ increased, - ─ decr.

There are five possible mechanisms by which lipid supplementation reduces methane: reducing fiber digestion (mainly in long chain fatty acids); lowering DMI (if total dietary fat exceeds 6-7%); suppression of methanogens (mainly in medium chain fatty acids); suppression of rumen protozoa and to a limited extent through biohydrogenation [42]. Oils offer a practical approach to reducing methane in situations where animals can be given daily feed supplements, but excess oil is detrimental to fiber digestion and animal production. Oils may act as hydrogen sinks but medium chain length oils appear to act directly on methanogens and reduce the numbers of ciliate protozoa. However, Kongmun et al. [55] reported that supplementation of coconut with garlic powder could improve *in vitro* ruminal fluid fermentation in terms of the volatile fatty acid profile, reduced methane losses and reduced protozoal population. Beauchemin et al. [42] recently reviewed the effects of the level of dietary lipid on methane emissions in 17 studies and reported that with beef cattle, dairy cows and lambs, there was a proportional reduction of 0.056 (g/kg DM intake) in methane for each 10 g/kg DM addition of supplemental fat. While this is encouraging, many factors need to be considered such as the type of oil, the form of the oil (whole crushed oilseeds vs. pure oils), handling issues (e.g. coconut oil has a melting point of 25°C) and the cost of oils which has increased dramatically in recent years due to the increased demand for food and industrial use. Few reports cover the effect of oil supplementation on methane emissions from dairy cows, where its impact on milk fatty acid composition and overall milk fat content would need to be carefully studied. Recent strategies, based on processed linseed, turned out to be very promising in both respects. Most importantly, a comprehensive whole system analysis needs to be carried out to assess the overall impact on global GHG emissions [45].

Manh et al. [56] reported that supplementation with Eucalyptus leaf meal at 100 g/d for ruminants could be an alternative feed enhancer: it reduces the production of rumen methane gas in cattle, while the digestibility of nutrients was unchanged. Conversely, Pilajun and Wanapat [54] reported that increasing the coconut oil and Mago-pel levels decreased proportion of methane production, and that a suitable level should not exceed 6% for coconut oil and 4% DM for MPP supplementation. In the future, comprehensive research into the individual components of essential oils, the physiological status of animals, the nutrient composition of diets and their effects on the rumen microbial ecosystem and metabolism of essential oils will be required to obtain consistent beneficial effects. Moreover, previous work, based on using plant secondary compounds and oils in both *in vitro* and *in vivo* trials, concerning rumen microorganisms, methane production and their impact on the mitigation of methane in the rumen, shows great potential for improving rumen ecology in the study of ruminant productivity (Table 9).

**Table 9:** Effects of plant secondary compounds and plant oil on digestibility and methane gas production in various studies

**Substrates**	**Level**	**Methane,%**	**Animal**	**References**
Garlic powder	16 mg	(−) 22.0*	Buffalo (fluid)	[55]
Coconut oil	16 mg	(+) 6.4*	Buffalo (fluid)	[55]
Soapberry fruit and mangosteen peel pellet	4%	10.0	Holstein heifers	[25]
Mangosteen peel powder	100 g/hd/d	(−) 10.5	Beef cattle	[51]
Coconut oil	7%	(+) 39.5*	Beef cattle	[51]
Coconut oil	7%	(−) 10.2*	Buffalo	[55]
Coconut oil Garlic powder	8:4 (mg)	(−) 18.9*	Buffalo	[55]
Coconut oil + Garlic powder	7% + 100 g	(−) 9.1*	Buffalo	[55]
Eucalyptus oil	0.33-2 ml/L	30.3-78.6%	Sheep	[57]
Eucalyptus oil	0.33-1.66 ml/L	4.47-61.0%	Buffalo	[58]
Eucalyptus meal leaf	100 g/d	reduce	Cow	[56]

* Values are significantly different (*P* < 0.05) from control group; +,- the values were increased or decreased from control group.

## Conclusion

We can conclude that local feed resources are of prime importance for ruminant feeding especially in the tropics and sub-tropical regions. These resources can be established, developed and utilized for feed on the farm as well as being processed commercially by industrial enterprises. They can be used as sources of energy and/or protein either as ingredients in concentrate mixtures or as feed supplements. They have provided good results for enriching the efficiency of rumen fermentation and subsequent ruminant productivity as well as mitigating rumen methane. Using feeds containing plant secondary compounds and essential oils is recommended as a means for reducing rumen methane. However, the potential benefits of manipulating rumen ecology to improve feed utilization efficiency in ruminants warrants undertaking further research and development in this area.

## Competing interests

The authors declare that they have no competing interests.

## Authors’ contributions

MW conceived of the manuscript’s purpose and design and critically revised the manuscript. SK and SP wrote and revised the manuscript according to MW’s suggestions. All authors read and approved the final manuscript submitted.
